# Static Behavior of Post-Installed High-Strength Large-Bolt Shear Connector with Fabricated Hybrid Fiber-Reinforced Concrete/Ordinary Concrete Deck

**DOI:** 10.3390/ma18051091

**Published:** 2025-02-28

**Authors:** Yuliang He, Junjie Li, Wujian He, Qiangqiang Wu, Yiqiang Xiang, Ying Yang

**Affiliations:** 1 College of Civil Engineering, Shaoxing University, Shaoxing 312000, China; hyliang@zju.edu.cn (Y.H.); 13216973620@163.com (J.L.); yangyingzju@126.com (Y.Y.); 2 College of Civil Engineering, Zhejiang University of Technology, Hangzhou 310000, China; 13735891670@163.com; 3 Zhejiang Communications Construction Group Co., Ltd., Hangzhou 310000, China; wuqiang_qiang@126.com; 4 College of Architectural Engineering, University of Zhejiang, Zhejiang 310027, China

**Keywords:** HFRC, push-out test, high-strength large bolt, capacity, slip behavior

## Abstract

Recent research indicates that high-strength bolts could be more effectively and efficiently used to connect steel girders and fabricated decks or retrofit existing composite girders than headed studs. To reduce the number of bolt shear connectors and, thus, further accelerate the construction of composite girders, high-strength large bolts could be an excellent alternative, resulting in greater concrete stress below the bolt. Also, hybrid fiber-reinforced concrete (HFRC) has better tensile ductility and strength than that of ordinary concrete (OC). Therefore, this study tried to design eighteen push-out test specimens, including different configurations of bolt shear connectors, to investigate the static properties of post-installed, high-strength, large-bolt shear connectors with fabricated HFRC/OC slabs. The experimental results indicated that the capacity and initial stiffness of a high-strength large through-bolt shear connector was the smallest. The fiber might enhance the capacity and initial stiffness of bolt shear connectors. Increasing the bolt diameter can significantly enhance the initial stiffness and load-bearing capacity, while the clearance of the bolt hole had a great influence on the capacity, initial stiffness, and slippage of the post-installed high-strength large-bolt shear connector. Finally, the capacity equation and slip behavior of post-installed, high-strength, large-bolt shear connector with fabricated HFRC deck were obtained using the regression method, which could provide the reference for their design.

## 1. Introduction

Compared with stud shear connectors, bolt shear connectors have become more and more popular for connecting the steel girder and fabricated deck of new composite structures or rehabilitating aging composite structures. This was mainly attributed to the fact that the bolt shear connector did not require welding, thus improving the fatigue strength and avoiding damage to the upper flange of the steel girder. A composite girder with bolt shear connectors could also be conveniently installed and disassembled. In recent years, experimental and numerical methods have been carried out to investigate the mechanical properties of bolt shear connectors in composite structures. Pathirana et al. [1] put forward an equation to evaluate the capacity of bolt shear connectors in grout and presented the flexural properties of composite beams with blind bolt shear connectors in grout using a numerical and experimental method. Henderson et al. [2,3] investigated the properties of composite beams retrofitted using various bolt shear connectors in grout through numerical and experimental methods. Chen et al. [4] presented the mechanical behavior of through-bolt shear connectors using push-out tests and numerical methods and developed a composite girder with through-bolt shear connectors. Liu et al. [5,6] presented the properties of high-strength friction-grip bolted shear connectors through numerical and experimental methods. Maio et al. [7] investigated the crack-induced degradation of the dynamic characteristics in plain concrete elements subjected to mixed-mode fracture conditions using numerical methods. Qi et al. [8] studied the shear strength of studs as shear connectors in steel–UHPC (ultra-high-performance concrete) composite structures through experimental methods. Lou et al. [9,10,11,12] investigated the performance of steel–concrete composite (EPSCC) beams through numerical and experimental methods. Zhang et al. [13] studied the performance of high-strength bolt shear connections with fabricated decks through experimental methods. Usually, the size of the bolt shear connector applied in a composite structure is typically 19 or 22 mm in diameter. To further reduce the number of holes cored in fabricated decks and the size of the grouted pockets connecting steel beams and fabricated decks, high-strength large bolts could be an excellent alternative. To the authors’ knowledge, little research has been found for high-strength large-bolt shear connectors (more than 22 mm in diameter) to date.

However, the higher stiffness and toughness of high-strength large-bolt shear connectors could easily cause pry-out or splitting failure in the concrete slab. To avoid such concrete failure, Sriboonma and Badie [14] advised adding the reinforcement ratio of rebars. In AISC [15], the minimum stud spacing was also suggested to prevent the phenomenon of stress concentration in the concrete slab. In recent years, high-strength concrete materials (high-strength concrete, fiber-reinforced cementitious composite, and so on) have been becoming more and more popular in steel–concrete composite structures to avoid brittle fracture. ACI [16] indicated that the incorporation of a certain amount of fibers (≤2% typically) into OC could limit the expansion of concrete cracks and greatly improve its fatigue resistance, impact resistance, durability, and post-cracking toughness. It was also found that various fibers incorporated together into concrete can further enhance its performance, which was termed hybrid fiber-reinforced concrete (HFRC) materials [17]. Recent research [18,19,20] showed that HFRC has relatively better performance than that of concrete reinforced by a single fiber and OC. To introduce HFRC (including steel fiber (SF) and polypropylene fiber (PF)) into the composite structure, the authors [21,22,23,24,25,26,27] replaced the conventional concrete slab with an HFRC slab and investigated the mechanical properties of stud/bolt connections in HFRC using 25 pushout test specimens and six composite girders. The results showed that stud/bolt connections in HFRC had better ductility and crack resistance and a slightly larger capacity than that in OC.

Owing to adding fibers, HFRC shows worse workability in comparison with OC. The transportation of HFRC is very difficult from the concrete mixer to the site by a transit mixer truck, and the pouring of HFRC is also not easy with a concrete pump on the construction site. To meet the requirement of transportation and pouring, the cost of improving the workability of HFRC is high. However, a potential way to solve these problems is for a fabricated HFRC deck to be used to replace the cast in situ HFRC deck and for post-installed, high-strength, large bolt shear connectors to be used to replace stud shear connectors. The fabricated HFRC deck can be produced in a prefabricated factory to omit the transporting link from the concrete mixer to the site and the pouring link in the construction site. Thus, steel girders and fabricated HFRC decks connected with post-installed high-strength large-bolt shear connectors could be applied widely in composite structures.

## 2. Methodology

This research attempted to take advantage of HFRC and high-strength large bolts. A novel post-installed, high-strength, large bolt shear connector was developed to transfer the shear force at the interface between the steel girder and the fabricated HFRC deck. Currently, there are no reports on the performance of post-installed, high-strength, large bolt shear connectors with fabricated HFRC decks. The authors attempted to investigate the composite action of the fabricated HFRC deck–girder interface with post-installed, high-strength, large bolt shear connectors through experimental methods and theoretical analysis. First, eighteen specimens were designed according to EC4 [28] to evaluate the interface interaction between the steel girder and the fabricated HFRC/OC deck using push-out test. Second, the influence of parameters, including the fibers, mortar strength, bolt diameter, single/double nut, and types of bolt shear connectors, on the performance of post-installed, high-strength, large bolt shear connectors with fabricated HFRC/OC decks was analyzed. Finally, equations for the slip behavior and ultimate capacity were proposed for the post-installed, high-strength, large bolt shear connectors with fabricated HFRC/OC decks using the regression method based on the test results.

## 3. Pushout Test

### 3.1. Fabrication of Test Specimens

**Figure 1:**
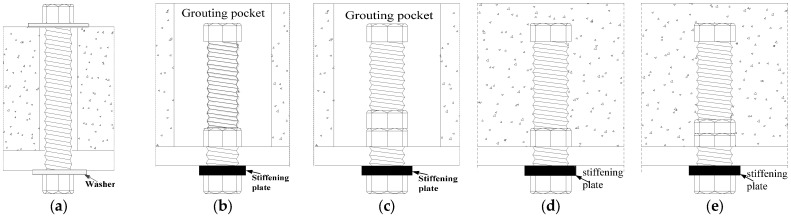
Configuration of high-strength large bolt shear connector. (**a**) Through bolt. (**b**) single nut with pocket. (**c**) double nuts with pocket. (**d**) single nut. (**e**) double nuts.

**Figure 2:**
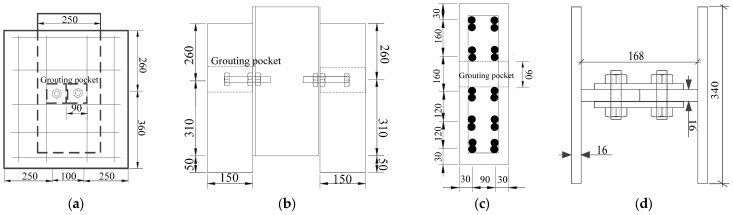
Test specimens (unit: mm). (**a**) Elevation view. (**b**) front view. (**c**) distribution of bar. (**d**) steel beam.

Figure 1 presents the configurations of post-installed, high-strength, large bolt shear connectors, which include three types: through-bolt (Figure 1a), single/double nut with grouting pocket (Figure 1b,c), and single/double nut embedded in HFRC/OC (Figure 1d,e). The connectors were made using 8.8-grade thread rods. To enhance the interaction between the concrete slab and the bolts, a hexagonal nut was added to the upper part of the threaded rod (Figure 1), similar to a stud head. The hexagonal nuts at the top and bottom of the HFRC/OC decks (Figure 1a) or steel flange (Figure 1b–e) were tightened with a preloading force of 5 kN using an electric torque-controlled wrench. Eighteen specimens were prepared to study the mechanical properties of post-installed, high-strength, large bolt shear connectors with fabricated HFRC/OC decks in accordance with EC4 [28]. The specimens were divided into 3 batches: through-bolt (NTPT1~2 and HTPT1~2), single/double nut with grouting pocket (NGPT1~4 and HGPT1~2), and single/double nut embedded in HFRC/OC (NPT1~4 and HPT1~4). Specimens HTPT1~2 and NTPT1~2 consisted of two fabricated HFRC/OC decks and a steel girder, with both sides of the steel girder connected to the decks by four bolts. For specimens HGPT1~2 and NGPT1~4, the steel girder was attached to the fabricated HFRC/OC decks using high-strength grout material and four high-strength large bolt shear connectors with a single/double nut, as shown in Figure 2a,b. The high-strength large bolts in specimens NPT1~4 and HPT1~4 were first embedded in the fabricated HFRC/OC decks and then connected to the steel girder using hexagonal nuts at the bottom of the steel flanges. The steel girder was composed of two steel T-girders, with a section of 340 mm × 200 mm × 16 mm × 16 mm (Figure 2d). The fabricated HFRC/OC decks had a thickness of 150 mm, a width of 600 mm, and a height of 620 mm. Two layers of reinforced bars (*Φ*12 mm) were placed in the HFRC/OC slabs to prevent splitting (Figure 2c). The high-strength through-bolt shear connectors (HTPT1~2 and NTPT1~2) were installed using precast holes in the fabricated HFRC/OC decks and the upper flange of the steel girder. Two diameters of precast holes were chosen for the fabricated HFRC/OC decks: 30 mm (for M27 8.8 bolts) and 33 mm (for M30 8.8 bolts). Additionally, four grouting pockets (90 mm × 90 mm) were reserved in the fabricated HFRC/OC decks for specimens HGPT1~2 and NGPT1~4. The clearance of the bolt holes in the steel girders was set to 1.5 mm. Considering that the capacity of the specimens could exceed the limit of the steel flange under the bolt holes, the steel flanges of specimens NPT4, HPT2~4, HGPT1~2, and NGPT1~4 were strengthened with stiffening plates, as shown in Figure 1b–e. The design parameters of specimens are listed in Table 1.

**Figure 3:**
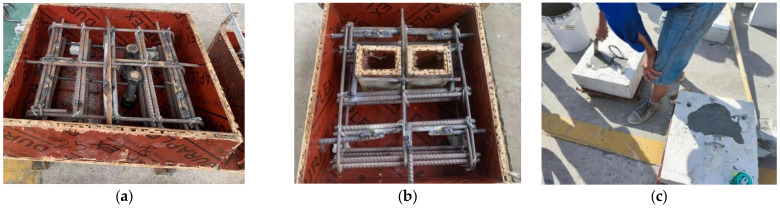
Fabrication of specimens. (**a**) NPT1~3, HPT1. (**b**) HGPT1~2 and NGPT1~4. (**c**) grouting.

In this study, fabricated HFRC/OC decks for all specimens were designed with a strength grade of approximately 50 MPa (28-day cubic compressive strength). Table 2 lists the mixture proportions of OC and HFRC. Before casting the concrete, the hexagonal nuts at the top and bottom of the formwork’s baseplate were tightened using an electric torque-controlled wrench to secure the high-strength large bolt shear connectors for specimens NPT1~4 and HPT1~4, as shown in Figure 3a. The bolt holes in the concrete slabs were prefabricated using PVC pipes with outside diameters of 30 mm and 33 mm for specimens HTPT1~2 and NTPT1~2, respectively. Additionally, 90 mm × 90 mm grouting pockets were reserved before casting specimens HGPT1~2 and NGPT1~4, as shown in Figure 3b. After 28 days, high-strength mortar material (with strength grades of C60 and C100) was poured into the grouting pockets, as shown in Figure 3c. All specimens were cast in a horizontal position to align with the construction of actual bridge decks. Cubic and prism specimens were cast simultaneously alongside the eighteen specimens and were cured under the standard conditions.

### 3.2. Material Properties

**Figure 4:**
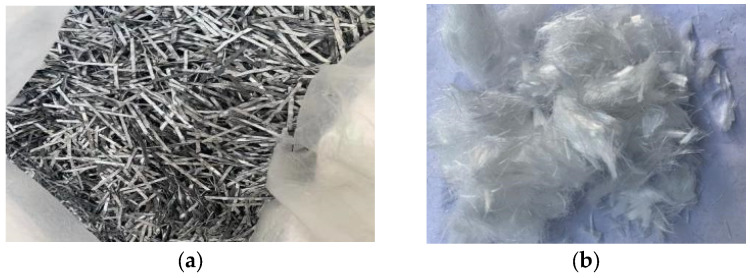
Schematic of fiber before mixing. (**a**) SF. (**b**) PF.

Recent research [16] found that fibers can significantly improve the inherent properties of OC with a relatively low volume (typically ≤2%). For SF, the most suitable volume proportion ranges from 0.5% to 2% to ensure an even distribution, while for PF, the optimal range is 0.05% to 0.2%. Chi et al. [18] demonstrated that HFRC with 1% SF and 0.15% PF can effectively leverage the material characteristics of both fibers. Based on this finding, the volume proportions of SF and PF used in this study were set at 1% and 0.15%, respectively, as shown in Figure 4. The physical properties of PF and SF, as provided by the manufacturer, are listed in Table 3.

The compressive strengths of concrete and mortar were determined through uniaxial compressive tests using 150 mm and 70.7 mm cubic specimens, respectively. The uniaxial tensile strength of mortar was measured using prism specimens (70.7 mm × 70.7 mm × 20 mm), while the uniaxial tensile strength of concrete was measured using larger specimens (150 mm × 150 mm × 460 mm). The 28-day elastic modulus of mortar was derived from the conversion of uniaxial cubic compressive strength, whereas the 28-day elastic modulus of concrete was obtained using a standard prism specimen (150 mm × 150 mm × 300 mm). The cylinder compressive strength of concrete was calculated by converting the cubic compressive strength, and the secant elastic modulus was determined by converting the elastic modulus at 28 days. The average values of the elastic modulus and tensile/compressive strength for both mortar and concrete are listed in Table 4. The performance properties of the bolts, steel plates, and reinforcing bars, as provided by the manufacturer, are listed in Table 5. The elastic modulus of the steel and bolts is 206 Gpa.

### 3.3. Test Loading Scheme

**Figure 5:**
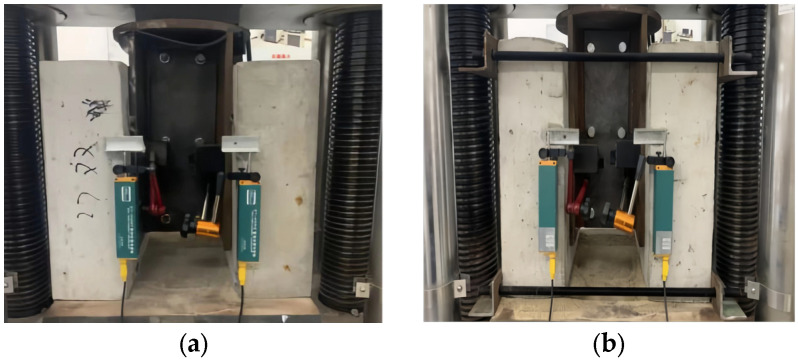
Loading of push out test specimen. (**a**) Non-reinforcement. (**b**) reinforced by tie rod.

Figure 5 shows the static load device with a capacity of 3000 kN. To ensure an even stress distribution on both sides of the specimens’ base, a thick steel plate was placed on the upper part of the steel girder, and a fine sand layer was laid on the lower part of the concrete slabs. To measure the slippage at the slab–girder interface, four linear variable displacement transducers (LVDTs) were arranged on both sides of each specimen, and their average values were recorded as the relative slip. To mitigate the influence of premature buckling of the steel flange around the bolt holes, some specimens (NPT4, HPT2~4, HGPT1~2, and NGPT1~4) were reinforced with tie rods, as shown in Figure 5b. Initially, a load step with an increment of 5 kN was applied from 0 to 100 kN at a speed of 0.2 kN/s to verify the proper functioning of the equipment. Next, the applied load was increased to 40% of the expected failure load (EFL) predicted by EC4 [28] at a speed of 0.5 kN/s. The loading cycle was repeated 25 times between 5% and 40% of the EFL to eliminate the initial slip caused by the bolt hole clearance. Subsequently, the load was increased from 0 to 60% of the EFL at a speed of 0.8 kN/s. Finally, a slip-controlled load step was applied at a speed of 0.5 mm/min and continued until failure.

## 4. Results and Discussion

### 4.1. Failure Pattern

**Figure 6:**
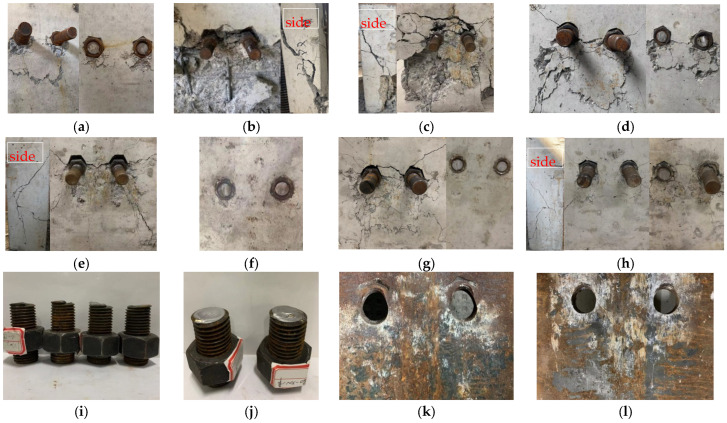
Failure of push-out test specimens NPT1~4 and HPT1~4. (**a**) NPT1 (OC slab). (**b**) NPT2 (OC slab). (**c**) NPT3 (OC slab). (**d**) NPT4 (OC slab). (**e**) HPT1 (HFRC slab). (**f**) HPT2 (HFRC slab). (**g**) HPT3 (HFRC slab). (**h**) HPT4 (HFRC slab). (**i**) HPT2 (bolt). (**j**) HPT3 (bolt). (**k**) NPT3 (steel beam). (**l**) NPT4 (steel beam).

The failure patterns of specimens NPT1~4 and HPT1~4 included three types, as shown in Figure 6. The first was that the bolt rods were sheared off on one side of the specimens NPT1, NPT4, and HPT3. Slight crushing and cracking of the concrete slabs were observed underneath the high-strength large bolts on the failure side. The second was the concrete slab failure occurred in specimens NPT2~3, HPT1 and HPT4, accompanied by obvious curvature at the base of the large bolts. Only one large bolt was sheared off in specimen HPT4. The third was the bolt shear connectors were sheared off simultaneously on both sides of specimen HPT2, indicating that both sides of the specimen had equal resistance. Specimen NPT1 experienced premature failure due to the bolt hole clearance, while the OC slabs remained intact (Figure 6a). Compared to specimens NPT2~3, the damage to specimens HPT1 and HPT4 was less severe when the concrete slabs failed, as shown in Figure 6b,c,e,h. This can be attributed to the higher ductility, better crack resistance, and higher compressive strength of HFRC. The crushing and cracking of concrete slabs underneath the high-strength large bolts were more severe in specimens with a single nut than in those with double nuts, as shown in Figure 6c,d,g,h. This indicates that double nuts can alleviate the stress concentration in the concrete slab underneath the high-strength large bolts. Only the damage to the OC slabs of specimen NPT2 was very severe. This might be because the steel girder of specimen NPT1 was reused for specimen NPT2 after local buckling occurred in the steel flange underneath the high-strength large bolt. The failure pattern of specimens NPT2~4 differed from those of specimens HPT2~4, which can be attribute to two factors. The first was that fibers improved the properties of the concrete, and the second was that specimens HPT2~4 were reinforced with tie rods during the loading process. Local buckling of the steel flange underneath the high-strength large bolts occurred in specimens NPT1~4 and HPT1~4. However, this buckling was significantly reduced after the steel flange was strengthened with stiffning plates around the high-strength large bolts, as shown in Figure 6k,l.

**Figure 7:**
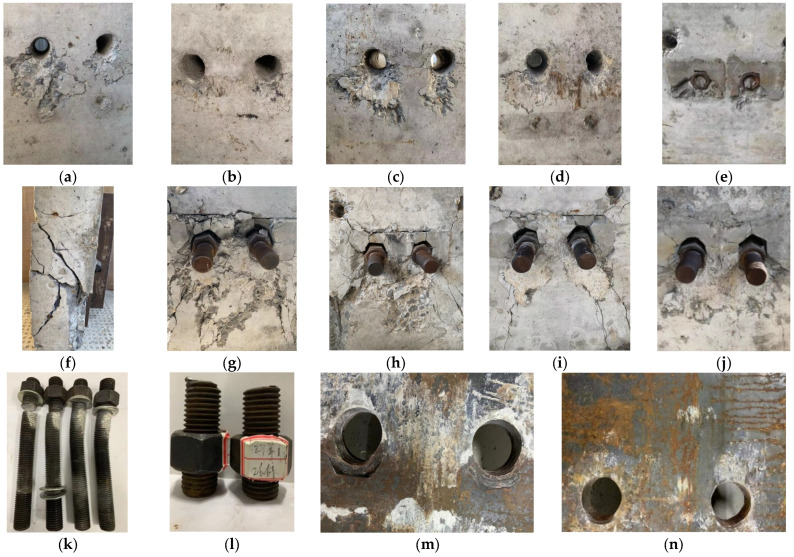
Failure mode of concrete slabs, steel flanges, and bolts for first/second groups. (**a**) NTPT1 (OC slab). (**b**) HTPT1 (HFRC slab). (**c**) NTPT2 (OC slab). (**d**) HTPT2 (HFRC slab). (**e**) NGPT1 (OC slab). (**f**) NGPT2 (OC slab). (**g**) NGPT3 (OC slab). (**h**) NGPT4 (OC slab). (**i**) HGPT1 (HFRC slab). (**j**) HGPT2 (HFRC slab). (**k**) though-bolt. (**l**) bolt with grouting pocket. (**m**) HGPT2 (steel beam). (**n**) HTPT2 (steel beam).

The failure modes of high-strength large bolts, steel girders, and concrete slabs for specimens NTPT1~2, HTPT1~2, HGPT1~2, and NGPT1~4 are shown in Figure 7. For specimens NTPT1~2 and HTPT1~2, the failure pattern involved one side of through-bolts being completely sheared off, with the root parts of the bolts exhibiting significant plastic deformation. No broken failure of the fabricated HFRC/OC slabs was observed for specimens NTPT1~2 and HTPT1~2. During the loading process, the bolt shank that was in the tightest contact with the bolt hole was sheared off first, followed by the sequential shearing of the remaining bolts until the specimens failed. For the second group, the fabricated concrete slabs failed in specimens NGPT2~4 and HGPT1~2, while one side of the bolts was sheared off in specimen NGPT1. Compared to specimens with OC slabs, the damage degree of specimens with HFRC slabs was less severe when the concrete slabs failed, as shown in Figure 6 and Figure 7a–d. The crushing and cracking of HFRC/OC slabs in specimens NPT1~4 and HPT1~4 (third group) were less severe than those in specimens with grouting pockets (second group) as shown in Figure 7e–j. This indicates that the coarse aggregate, fibers and rebar helped alleviate the stress concentration in the concrete slabs underneath the high-strength large bolts. Only the damage degree of the OC slab was very slight for specimen NGPT1 with a single nut. The might be attributed to the nearly equal resistance on both sides of specimen NGPT1, resulting in the simultaneous shearing of all four high-strength large bolts. The splitting of the OC slabs was very severe, and dismantling was difficult for specimen NGPT2 with a double nut, as shown in Figure 7f. This finding contradicts Yang et al. [29], who suggested that double nuts could delay the crushing and cracking of concrete under bolt shear connectors. The discrepancy may be due to manufacturing errors in the bolt holes. Local buckling of the steel flange underneath the high-strength large bolts occurred in specimens NTPT1~2, HTPT1~2, HGPT1~2, and NGPT1~4. Splitting cracks on the sides of the concrete slabs were observed in both groups, but these cracks were significantly reduced in specimens reinforced with fibers. Kim et al. [30] reported that shear connectors failed when the concrete strength grade exceeded 40 MPa, while the concrete slab failed when the concrete strength grade was 30 Mpa or less. However, the test results in this study were inconsistent with the findings of Kim et al. [30], except for the first group. This discrepancy might be attributed to two factors; first, the capacity of all specimens was very high, exceeding the limit of the HFRC/OC slabs and steel flanges, and second, the clearance of the bolt holes had a significant influence on the damage degree of the HFRC/OC slabs.

### 4.2. Load–Slip Curve

**Figure 8:**
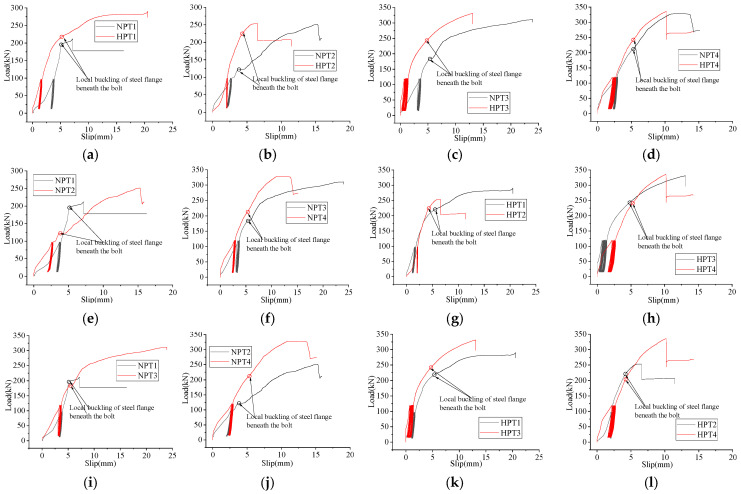
Load–slip curve of third group. (**a**) NPT1, HPT1. (**b**) NPT2, HPT2. (**c**) NPT3, HPT3. (**d**) NPT4, HPT4. (**e**) NPT1, NPT2. (**f**) NPT3, NPT4. (**g**) HPT1, HPT2. (**h**) HPT3, HPT4. (**i**) NPT1, NPT3. (**j**) NPT2, NPT4. (**k**) HPT1, HPT3. (**l**) HPT2, HPT4.

**Figure 9:**
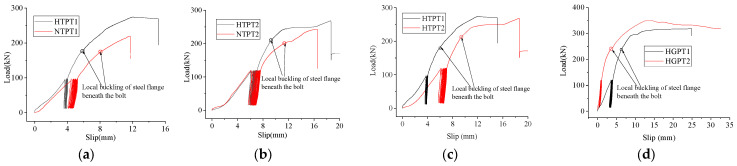

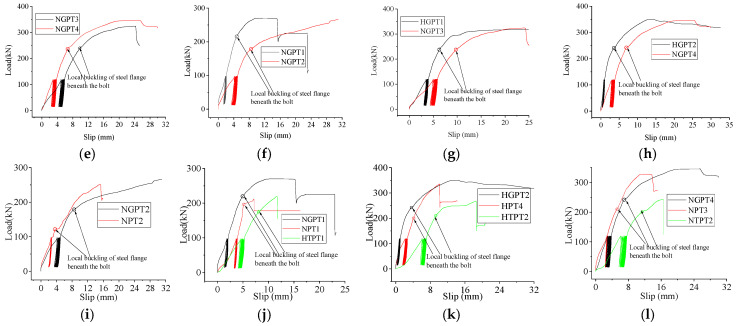
Comparison of load–slip curve. (**a**) HTPT1, NTPT1. (**b**) HTPT2, NTPT2. (**c**) HTPT1, HTPT2. (**d**) HGPT1, HGPT2. (**e**) NGPT3, NGPT4. (**f**) NGPT1, NGPT2. (**g**) HGPT1, NGPT3. (**h**) HGPT2, NGPT4. (**i**) NGPT2, NPT2. (**j**) NGPT1, NPT1, NTPT1. (**k**) HGPT2, HTPT2, HPT4. (**l**) NTPT2, NPT3, NGPT4.

When an external load was applied to all specimens, slip occurred between the fabricated HFRC/OC deck and the steel girder. The slip behavior of the post-installed, high-strength, large bolt shear connector is shown in Figure 8 and Figure 9. Initially, all specimens were subjected to repeated loading to eliminate the initial slip between the steel girder and the fabricated HFRC/OC deck caused by the clearance of the bolt holes. During this phase, the initial shear stiffness was very small. However, the shear stiffness increased significantly after 25 cycles. As the load increased, the load–slip curve began to enter a nonlinear phase. Due to manufacturing errors in the bolt holes of the fabricated HFRC/OC decks or steel girders, the distribution of bolt hole clearance was uneven around the bolt shank for each post-installed, high-strength, large bolt shear connector. This resulted in varying degrees of contact between the bolt hole and the bolt shank. When the failure mode involved the bolt being sheared off, the bolt that was in the tightest contact with the bolt hole was the first to fail, causing a sharp drop in shear stiffness, as shown in Figure 8 and Figure 9. Conversely, when the fabricated HFRC/OC decks failed, the concrete in closest contact with the high-strength large bolt cracked and crushed first, leading to a slower decline in shear stiffness. The slip width after 25 cycles represented the residual slippage caused by the bolt hole clearance. This residual slippage was determined by the manufacturing errors in the bolt holes and ranged from 1.5 mm to 3 mm in this study.

The slip behavior of the high-strength large bolt shear connector in HFRC/OC is shown in Figure 8 and includes five phases: the initial slipping phase, cyclic phase, elastic phase, strengthening phase, and descent phase. In the initial state, the load–slip curves are highly irregular due to the significant influence of the bolt hole clearance on slippage. After cyclic loading, the residual slip caused by the bolt hole clearance was reduced, and the load–slip curve began to enter the elastic phase. As the load increased, the HFRC/OC slabs cracked and crushed underneath the high-strength large bolts. Shortly afterward, local buckling of the steel flange occurred below the high-strength large bolts, and the load–slip curve became nonlinear. The stiffness then decreased rapidly, and the slip continued to increase. The specimens entered the descent stage when the load reached the ultimate capacity of the bolt shear connector. When the failure mode involved the high-strength large bolts being sheared off, the bolt in the tightest contact with the bolt hole was the first to fail, causing the load–slip curve to drop sharply and a platform to appear. As the load continued to be applied, the next bolt in the tightest contact with the bolt hole among the remaining bolts was sheared off again. The test was terminated once one side of the two bolts was completely sheared off. However, no platform appeared when the failure mode involved the concrete slab breaking.

For specimen NPT1, local buckling of the steel flange began to occur underneath the high-strength large bolt when the load reached 782 kN. Subsequently, the shear stiffness decreased slowly, and a yield platform appeared. When the load reached 887.2 kN, the first bolt was sheared off, causing the load to drop sharply, and another platform appeared. The second bolt on the same side was then sheared off during unloading, leading to the failure of specimen NPT1. The slip behavior of specimen HPT2 was similar to that of specimen NPT1 due to their comparable failure patterns. For specimens HPT1 and NPT2-3, the failure pattern involved the splitting of the concrete slab. After local buckling of the steel flange occurred below the high-strength large bolts, the strengthening phase was longer compared to that of specimens NPT1 and HPT2. For specimen HPT4, the load dropped sharply after the first bolt was sheared off, accompanied by a yield platform. In specimen NPT4, two bolts on the same side were sheared off simultaneously, and a platform also appeared. As shown in Figure 7, the incorporation of fibers may improve the capacity and stiffness of the high-strength large bolt shear connector, as well as the local buckling capacity of the steel flange. Figure 7e,h indicate that the influence of single or double nuts on the slip behavior was inconsistent. The local buckling capacity of the steel flange for specimen NPT1 with a single nut was significantly higher than that of specimen NPT2 with double nuts, while the opposite was true for specimens NPT3-4. However, after fibers were added to the concrete, the local buckling capacity of the steel flange for specimens with a single nut became comparable to that of specimens with double nuts. According to Figure 7i–l, the local buckling capacity of the steel flange showed little correlation with the bolt diameter. Instead, the degree of contact between the bolt hole and the bolt shank had a significant influence on the local buckling capacity of the steel flange.

The slip behavior of the high-strength large through-bolt shear connector with nominal diameters of 27 mm and 30 mm is shown in Figure 9a–c. The slip behavior of the four specimens (HTPT1~2 and NTPT1~2) was similar. Since PF and SF were incorporated into the concrete slabs of specimens HTPT1~2, their initial stiffness was greater than that of specimens NTPT1~2. The shear stiffness of specimens NTPT1~2 declined faster than that of specimens HTPT1~2 after yielding began. This indicates that HFRC may improve the shear stiffness of the large through-bolt shear connector. When the load per bolt reached 175 kN for specimens NTPT1 and HTPT1, the steel flange underneath the high-strength large bolts began to yield. The corresponding load per bolt was 205 kN for specimens NTPT2 and HTPT2. After the steel flange yielded, the shear stiffness decreased more rapidly, with a slight increase in load. As the applied load approached the ultimate capacity of the bolt shear connector, a yield platform appeared for specimens HTPT1~2. This suggests that fibers improved the ductility of the high-strength large through-bolt shear connector. The first bolt was sheared off when the load reached the ultimate capacity of the bolt shear connector, causing the load–slip curve to drop sharply. Subsequently, the second bolt on one side was also sheared off, leading to the failure of the specimen. The stiffness and ultimate capacity of specimen HTPT2, with a nominal diameter of 30 mm, were lower than those of specimen HTPT1, with a nominal diameter of 27 mm. This was due to manufacturing errors in the bolt hole, which caused premature failure in specimen HTPT2. The local buckling capacity of the steel girder for specimens NTPT2 and HTPT2 was slightly greater than that of specimens NTPT1 and HTPT1, owing to the larger contact area between the bolt hole and the bolt shank.

The slip behavior of the high-strength large bolt shear connector with the grouting pocket is shown in Figure 9d–h. The curves exhibited an obvious yield platform as the applied load approached the capacity of the bolt shear connector. The yield platform was the shortest for specimen NGPT1 because the failure mode involved the bolts being sheared off. Due to manufacturing errors in the bolt hole, the shear stiffness of specimen NGPT1 with single nuts was greater compared to specimen NGPT1 with double nuts. After fibers were incorporated into the concrete slabs, the stiffness and ductility of specimens HGPT1~2 were greater than those of specimens NGPT3~4, as shown in Figure 9g and h. Compared to specimens HGPT1 and NGPT3, the shear stiffness and ductility of specimens HGPT2 and NGPT4 were enhanced due to the use of higher-grade mortar, as shown in Figure 9d,e. When the load per bolt approached 240 kN for specimens NGPT3~4 and HGPT1~2, the steel flange underneath the high-strength large bolts began to yield. The corresponding load per bolt was 219 kN and 178 kN for specimens NGPT1 and NGPT2, respectively.

A comparison of the slip behavior among the high-strength large through-bolt shear connector, the high-strength large bolt shear connector in HFRC/OC, and the high-strength large bolt shear connector with grouting pockets is shown in Figure 9i–l. The yield and descent segments of the curves for specimens NGPT1~2, NGPT4, and HGPT2 were longer than those of specimens HTPT1~2, NTPT2, NPT1~3, and HPT4. This indicates that the ductility of the high-strength large bolt shear connector with grouting pockets was the greatest among the three groups. The initial stiffness of the high-strength large through-bolt shear connector was the smallest among the three groups, as evident from the initial slipping phase in Figure 9j–l. The initial stiffness of specimens NPT1 and HPT1 was lower compared to specimens NGPT1 and HGPT2, while the initial stiffness of specimens NGPT2 and NGPT4 was smaller compared to specimens NPT2-3. This suggests that the failure mode had a significant effect on the initial stiffness. The local buckling capacity of the steel girder for the high-strength large bolt shear connector with grouting pockets was greater than that of the high-strength large bolt shear connector in HFRC/OC, as shown in Figure 9i–l. The reason for this might be that the early crushing and cracking of the mortar, caused by stress concentration under the high-strength large bolts, reduced the non-uniform distribution of shear force resulting from the bolt hole clearance among the four bolts. This was due to the absence of rebar and coarse aggregate in the mortar.

The initial stiffness, slippage, and capacity of specimens NPT1~4 and HPT1~4 are listed in Table 6. The initial stiffness was expressed as the ratio of 0.5Pu (maximum load) to the corresponding slippage, according to Yang et al. [29]. It was found that there was no correlation between the initial stiffness and factors such as fibers, bolt diameter, or single/double nuts, which aligns with the findings of Yang et al. [29]. When the residual slippage after 25 cycles was deducted from the corresponding slippage at 0.5Pu, the data in the sixth row of Table 6 indicate that fibers, bolt diameter, and double nuts might enhance the shear stiffness. It was also evident that the clearance of the bolt hole had a significant influence on the initial stiffness of the high-strength large bolt shear connector in HFRC/OC, as shown by the data in the fifth row of Table 6. According to EC4 [28], the slippage must exceed 6 mm to redistribute the shear at the slab–girder interface. The ultimate slippage of specimens NPT1~4 and HPT1~4 exceeded 6 mm. The ultimate slippage was greater when the concrete slab failed in specimens NPT2~3 and HPT1. Conversely, the ultimate slippage was smaller when the high-strength large bolts were sheared off in other specimens. After the steel flange was strengthened, the ultimate slippage decreased significantly for specimens HPT2~4. The fourth row of Table 6 shows that there was little correlation between the ultimate slippage and factors such as fibers, single/double nuts, or the bolt diameter. The ultimate slippage of the bolt shear connector with a coupler [29] was less than 6 mm due to the use of a thicker concrete slab (500 mm) and steel flange (30 mm). In contrast, the ultimate slippage of the through-bolt shear connector [13] exceeded 6 mm because of the larger bolt hole clearance. This suggests that increasing the bolt hole clearance or reducing the thickness of the steel flange may enhance the ultimate slippage to meet the requirements of EC4 [28]. Table 6 also shows that the ultimate capacity increased as the bolt diameter increased. Based on the test results of specimens NPT3~4 and HPT3~4, fibers and double nuts might slightly enhance the capacity of the high-strength large stud shear connector in HFRC/OC, which is consistent with the findings of Yang et al. [29] and He et al. [25].

The initial stiffness, ultimate capacity, and ultimate slippage for specimens NTPT1~2, HTPT1~2, HGPT1~2, and NGPT1~4 are listed in Table 7. The ultimate slippage showed little correlation with variations in mortar strength, bolt diameter, or fibers. The average ultimate slippage for the three groups was 15.46 mm, 21.67 mm, and 13.54 mm, respectively, as listed in Table 6 and Table 7. This indicates that the ductility of the high-strength large bolt shear connector with grouting pockets was the best among the three groups, while the ductility was the smallest for the high-strength large bolt shear connector in HFRC/OC. After 25 cycles of loading, the shear stiffness improved significantly and increased with higher mortar strength and larger bolt diameter. Fibers may also enhance the shear stiffness of post-installed, high-strength, large bolt shear connectors, which is consistent with the findings of He et al. [25]. For specimens with a nominal diameter of 27 mm, the capacity of the high-strength large bolt shear connector with grouting pockets was the highest among the three groups, while the capacities of the other two groups were comparable. However, for specimens with a nominal diameter of 30 mm, the capacity of the high-strength large through-bolt shear connector was the lowest among the three groups, while the capacities of the other two groups were comparable. As the mortar strength and bolt diameter increased, the capacity of the post-installed, high-strength, large bolt shear connector increased slightly. Fibers may also slightly improve the capacity of the post-installed, high-strength, large bolt shear connector, as shown in Table 6 and Table 7.

## 5. Evaluation of Test Results

The ultimate capacity and slip behavior are two key indicators that reflect the mechanical properties of shear connectors. The ultimate capacity determines the number of shear connectors required, while the load–slip curve illustrates the stiffness and ductility of the shear connectors in the design of composite structures. These properties are primarily obtained through FE analysis or push-out tests. Regression analysis is then conducted to derive fitting coefficients, enabling the formulation of empirical equations for the ultimate capacity and slip behavior. Currently, the equations of the ultimate capacity and slip behavior of stud shear connectors are well established. However, to the authors’ knowledge, limited research has been conducted on the ultimate capacity and slip behavior of post-installed, high-strength, large bolt shear connectors. In fact, the ultimate capacity and slip behavior of bolt shear connectors differ from those of stud shear connectors due to the influence of the bolt hole clearance, although their working mechanisms are similar in transferring shear at the slab–girder interface. Based on equations recommended in previous studies, test results from the literature, and experimental data presented in this paper, the authors attempted to derive equations for the capacity and slip behavior of post-installed, high-strength, large bolt shear connectors with fabricated HFRC/OC decks using regression analysis.

### 5.1. Capacity of Post-Installed, High-Strength, Large Bolt Shear Connectors

At present, there is no established code for calculating the capacity of post-installed, high-strength, large bolt shear connectors with fabricated HFRC/OC decks. The equations provided in current specifications are primarily applicable to evaluating the capacity of stud shear connectors. Push-out tests have revealed that the behavior of post-installed, high-strength, large bolt shear connectors in fabricated HFRC/OC decks connected to steel girders differs from that of stud shear connectors due to the influence of the bolt hole clearance. The authors of [25] previously derived a capacity equation for through-bolt shear connectors with fabricated HFRC/OC decks based on ten push-out test specimens featuring small bolt diameters (less than 22 mm). This equation took into account the effects of PF (precast factor) and SF (slip factor). Zhang et al. [13] used seventeen FE models and eleven push-out tests to develop capacity equations for through-bolt shear connectors. Similarly, Zhao et al. [31] proposed a formula for through-bolt shear connectors that incorporates the contributions of both the bolt and the surrounding concrete. Table 8 summarizes the capacity equations for through-bolt shear connectors recommended by various studies. Based on the equations listed in Table 8 and the test results obtained in this study, the authors also attempted to derive a capacity equation for high-strength large through-bolt shear connectors with fabricated HFRC/OC decks.

Based on variance analyses and test results, Chi et al. [18] proposed Equation (1) to estimate the uniaxial compressive strength of HFRC. Huang [32] found that the elastic modulus of HFRC was solely related to the volume fraction of SF, as expressed by Equations (2) and (3).

(1) $$f_{fc}=\left(1+0.388\lambda_{pf}+0.206\lambda_{sf}\right)f_{c}$$

(2) $${f^{\prime }}_{fc}=f_{c}\left(1+0.056\lambda_{sf}\right)$$

(3) $$E_{fc}=\frac{10^{5}}{2.2+34.74/{f^{\prime }}_{fc}}$$

**Figure 10:**
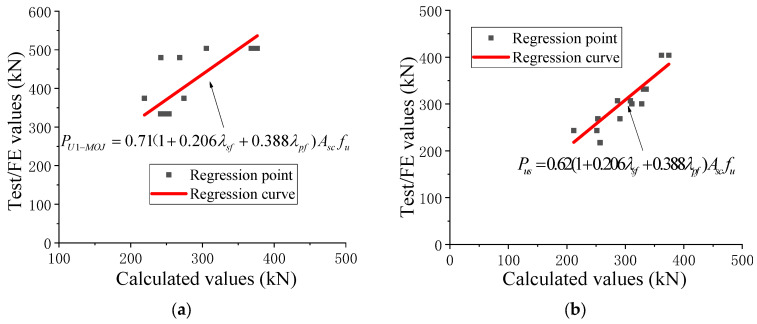
Regression curve of coefficient. (**a**) Though-bolt shear connector. (**b**) bolt shear connector in HFRC/OC.

Generally, the equations used for evaluating the capacity of stud shear connectors include two cases: concrete failure and stud failure. In Table 8, Zhang et al. [13] and He et al. [25] proposed capacity equations for the two failure modes of through-bolt shear connectors, respectively. Zhao et al. [31] also considered both failure modes but provided only one equation. In this study, all specimens with high-strength large through-bolt shear connectors failed due to bolt failure. Therefore, to derive the capacity equation for post-installed high-strength large through-bolt shear connectors with fabricated HFRC/OC decks, this study focused solely on bolt failure. A fitting formula (Equation (4)) was directly established based on the work of He et al. [25]. The coefficient aa was determined to be 0.71 through a regression analysis of experimental results from Zhang et al. [13] (NC-1~2 and SFRC-1~7) and the results presented in this paper (Figure 10a).

(4) $$P_{U1-MOJ}=\alpha (1+0.206\lambda_{sf}+0.388\lambda_{pf})A_{s}f_{u}$$

Table 9 lists the ratio of the test results to the calculated values. The ultimate capacities of post-installed high-strength large through-bolt shear connectors with fabricated HFRC/OC decks, as proposed by He et al. [25], were, on average, 26% smaller than the test results for concrete failure and 48% smaller for bolt failure. The coefficients of variation were relatively high (11.8% for concrete failure and 11.4% for bolt failure). This discrepancy might be attributed to the lower bolt strength in He et al. [25] compared to that found in Zhang et al.’s work [13] and this study. The equation proposed by Zhang et al. [13] overestimated the ultimate capacity by an average of 10% for concrete failure and only 3% for bolt failure. The coefficient of variation was lower (7.8%) for concrete failure but higher (14.5%) for bolt failure. This difference might be due to the fact that the bolt hole clearance was filled with high-strength grouting material. The ultimate capacities predicted by Zhao et al. [31] also overestimated the experimental results by an average of 8%, as shown in Table 9, with a coefficient of variation of only 8.4%. This can be attributed to the inclusion of the concrete contribution in the equation proposed by Zhao et al. [31]. It was found that the capacities calculated using Equation (4) were consistent with the experimental results, indicating that the equation proposed in this paper provides more accurate results compared to those listed in Table 8. The CV for Equation (4) was the lowest among all the equations. The test results demonstrated that the equations proposed in this paper can be effectively used to evaluate the capacity of post-installed high-strength large through-bolt shear connectors with fabricated HFRC/OC decks.

According to Kim et al. [30], the threshold between the two failure patterns of stud shear connectors typically lies within a concrete compressive strength range of 30–40 MPa. This suggests that the failure pattern of high-strength large bolt shear connectors in HFRC/OC is likely to be the shearing of high-strength large bolts, while concrete failure might be triggered by the presence of bolt hole clearance. Table 10 presents the equations proposed by Zhang et al. [33], Pathirana et al. [1], and Yang et al. [29], which provide two equations: one for concrete failure and the other for bolt failure. In this study, the compressive strength of the HFRC/OC deck exceeded 50 MPa, placing the high-strength large bolt shear connectors in HFRC/OC clearly beyond the scope of these equations. Therefore, whether the research findings summarized in Table 10 can be accurately applied to predict the capacity of high-strength large bolt shear connectors requires further verification.

Pathirana et al. [1] and Zhang et al. [33] considered two failure modes and modified the capacity equations in EC4 [28] to derive the capacity equations for bolt shear connectors. In contrast, Yang et al. [29] only considered bolt failure, as shown in Table 10. To develop the capacity equation for high-strength large bolt shear connectors in HFRC/OC, this paper also attempted to account for both failure modes. Based on the experimental results of specimens NPT1~4 and HPT1~4, the fibers were found to slightly improve the capacity of high-strength large bolt shear connectors. The authors [24,25] also observed that shear connectors in OC had a slightly lower capacity compared to those in HFRC. To incorporate the influence of fibers on the capacity of high-strength large bolt shear connectors in HFRC/OC under bolt failure conditions, Equation (4) was applied to determine the fitting parameter a through regression analysis of push-out test results from this study and that by Yang et al. [29] (B7~9). The resulting equation (Equation (5)) of the capacity of high-strength large bolt shear connectors in HFRC/OC is presented in Figure 10b.

(5) $$P_{U2-MOJ}=0.62(1+0.206\lambda_{sf}+0.388\lambda_{pf})A_{sc}f_{u}$$

Strictly speaking, the experimental results indicated that the failure pattern of the specimens in this study was a mixed mode, involving both localized crushing and cracking of the concrete underneath the bolt and bolt failure. For specimens NPT2-3, HPT1, and HPT4, although the primary mode was concrete failure, the damage to the high-strength large bolts was also severe. He et al. [23,24,25,26] previously established the relationships between the uniaxial compressive strength and elastic modulus of HFRC and OC, respectively. When the concrete slab failed, this study proposed equations (Equations (6) and (7)) to predict the capacity of high-strength large bolt shear connectors in HFRC. These equations were derived by modifying Equation (7) in work by Pathirana et al. [1] and Equation (6) in work by Zhang et al. [33], as presented in Table 10. In these modified equations, the elastic modulus and uniaxial compressive strength of HFRC were used in place of those of OC.

(6) $$P_{U3-MOJ}=0.7A_{SC}\sqrt{f_{fc}E_{fc}}$$

(7) $$P_{U4-MOJ}=0.31d_{SC}^{2}\sqrt{f_{fc}E_{fc}}$$

Table 11 lists the comparison of the results obtained from the tests and the recommended equations. The ultimate capacities (*P_us_*) of bolt shear connectors in HFRC/OC, as predicted by Yang et al. [29] and Pathirana et al. [1], were, on average, 17% higher than the experimental results, providing non-conservative estimates. In contrast, Zhang et al. [33] provided conservative results. For concrete failure, the shear capacities (*P_uc_*) recommended by Pathirana et al. [1] were, on average, 25% lower than the experimental results, while those from Zhang et al. [33] overestimated the shear capacity by an average of 13%. These findings indicate that the equations listed in Table 10 are not suitable for estimating the shear capacities (*P_us_*) of high-strength large bolt connectors in HFRC/OC. Regarding the equations proposed in this study, the shear capacities (*P_u3–MOJ_*) derived from Equation (6) were, on average, 13% higher than the experimental results, significantly overestimating the shear capacity of bolt shear connectors in HFRC/OC. Conversely, the shear capacities (*P_u4–MOJ_*) from Equation (7) underestimated the capacity by an average of 26% compared to the experimental results. In contrast, the shear capacities (*P_u2–MOJ_*) from Equation (5) showed good agreement with the test results, demonstrating greater accuracy compared to the equations in Table 10. The CV for *P_u2–MOJ_* was only 0.061, as listed in Table 11. These results suggest that Equation (5) has potential for evaluating the shear resistance of high-strength large bolt shear connectors in HFRC/OC.

According to the push-out test results, the shear capacity of high-strength large through-bolt shear connectors was the smallest among the three groups, while the shear capacity of high-strength large bolt shear connectors in HFRC/OC was comparable to that of high-strength large bolt shear connectors with grouting pockets. To date, the shear capacity of bolt shear connectors with grouting pockets cannot be obtained from existing codes or references. Therefore, this paper attempted to select a suitable equation from Table 10 and Equations (5) and (6).

Table 12 lists the comparison of the calculated values and experimental results for high-strength large bolt shear connectors with grouting pockets. When HFRC/OC decks failed, the ultimate capacities (*P_uc_*) of bolt shear connectors predicted by Pathirana et al. [1] and Equation (7) were, on average, 38% lower than the experimental results, providing conservative estimates. In contrast, the calculated values from Equation (6) and Zhang et al. [33] yielded non-conservative results. In terms of bolt failure, the ultimate capacities (*P_us_*) recommended by Pathirana et al. [1] and Equation (5) were, on average, 13% and 5% lower than the test results, respectively. The calculated values from Zhang et al. [33] and Yang et al. [29] overestimated the ultimate capacity by an average of 12% and 6%, respectively, compared to Equation (5). The results demonstrated that the shear capacities (*P_u2–MOJ_*) derived from Equation (5) agreed well with the test results, indicating greater accuracy compared to the equations in Table 10. The CV for *P_u2–MOJ_* was only 0.051, as listed in Table 12. These findings suggest that the empirical Equation (5) has the potential for estimating the shear capacity of high-strength large bolt shear connectors with grouting pockets.

### 5.2. Load–Slip Curve

**Figure 11:**
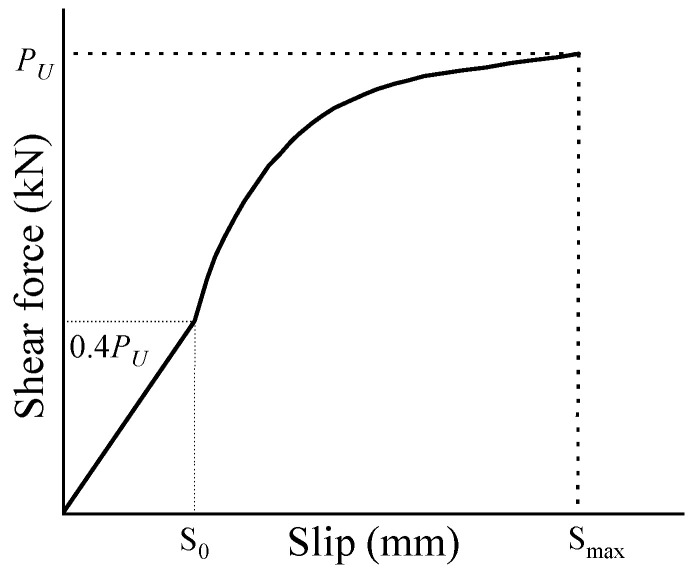
Slip model.

The static behavior of shear connectors can be represented by the load–slip curve, which reflects characteristics such as the initial stiffness and ductility of the connector. To date, the investigation of slip behavior is well-established for stud shear connectors. However, to the authors’ knowledge, there is limited research on the slip behavior of post-installed, high-strength, large bolt shear connectors. In fact, the slip behavior of bolt shear connectors differs from that of stud shear connectors due to the presence of the bolt hole clearance. Yang et al. [29] derived a formula for the slip behavior of bolt shear connectors through single-parameter fitting analysis and established a linear relationship between the single parameter and the bolt hole clearance using regression analysis. They found that the slip behavior of bolt shear connectors aligned with that of stud shear connectors. Liu et al. [5] suggested that the load–slip curve of bolt shear connectors should be divided into three distinct regimes: (1) when friction was the primary mechanism for transferring shear force at the slab–girder interface, the slip was nearly zero; (2) after the interface friction was overcome, the relationship between load and slippage became linear; and (3) when the load exceeded *Q*_0_ (maximum friction) + 20 kN, the load–slip curve followed an exponential function, similar to that of stud shear connectors. Some studies [34] have shown that the non-uniform distribution of shear force at the slab–girder interface can affect the mechanical behavior of composite structures, and a reduction in shear stiffness may alleviate this non-uniformity. The bolt hole clearance could serve as an effective way to reduce the shear stiffness of bolt shear connectors. However, applying pretension to bolt shear connectors might not be advisable, as it significantly increases the initial stiffness. Therefore, this study recommends avoiding the use of pretension in high-strength large bolt shear connectors. Instead, the load–slip curve is divided into two distinct phases: a linear phase and a single-parameter exponential phase, as shown in Figure 11. The equations for the load–slip curve (Equations (8) and (9)) were derived through a regression analysis of the experimental results, taking into account the effects of SF and PF.

(8) $$P/P_{u}=(0.03+0.206\lambda_{sf}+0.388\lambda_{pf})S\;\;\;\;\;\;\;\;\;\;\;\;\;\;\;(P\;\mathrm{less}\;\mathrm{than}\;0.4P_{u})$$

(9) $$P/P_{u}=1-e^{(-0.45+0.206\lambda_{sf}+0.388\lambda_{pf})S}\;\;\;\;\;\;\;\;\;\;\;\;(P\;\mathrm{more}\;\mathrm{than}\;0.4P_{u})$$

(10) $$P/P_{u}={\left(1-e^{-0.2S}\right)}^{0.4}$$

(11) $$P/P_{u}=1-e^{-As}\;\;\;\;\;\;\;\;(A=4.03c-2.48)$$

(12) $$P/P_{u}=(S/d)/(0.006+1.02S/d)$$

where *P_u_* is the measured maximum load per bolt (kN), *S* is the slippage, *d* is the bolt diameter, and *P* is the applied load of the bolt (kN).

**Figure 12:**
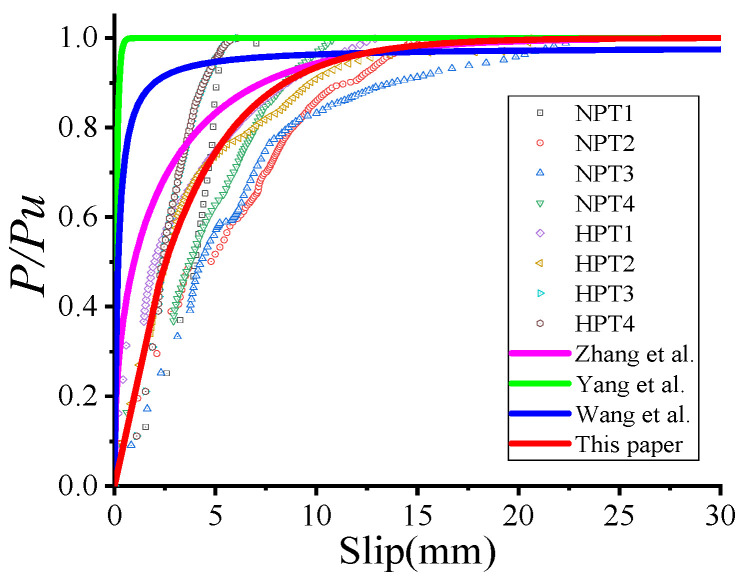
Load–slip curve of bolt connector in HFRC/OC. Zhang et al. [27]. Yang et al. [23]. Wang et al. [29].

**Figure 13:**
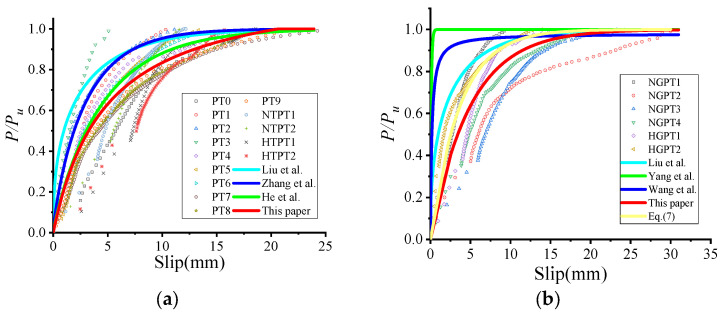
Load–slip curve. (**a**) Though-bolt shear connector. (**b**) bolt shear connector with grouting pocket. Liu et al. [5]. Zhang et al. [7]. He et al. [19]. Yang et al. [23]. Wang et al. [29].

The comparison of load–slip curves obtained from the tests and the recommended equations is shown in Figure 12. Equation (10) was used to describe the slip behavior of bolt shear connectors in steel-fiber (SF) reinforced concrete [31], Equation (11) was used for a novel bolt shear connector [29], and Equation (12) was used for large stud shear connectors in ultra-high performance concrete (UHPC) [35]. Equations (10)–(12) underestimated the ratio of slippage to *P*/*P_u_* for the test specimens (Figure 9). This discrepancy might be due to the smaller ratio of slippage to *P*/*P_u_* observed in the test specimens compared to the ratios predicted by the empirical equations. Equation (10) was developed for steel–SF reinforced concrete composite girders, where the bolt hole clearance was filled with high-strength mortar, resulting in a smaller ratio of slippage to *P*/*P_u_*. Equation (11) was applied to novel bolt shear connectors connected to very thick OC slabs (500 mm) and steel flanges (30 mm), leading to a reduced slippage per *P*/*P_u_* ratio. Meanwhile, Equation (12) described the slip behavior of large stud shear connectors in UHPC, where the combination of high-strength concrete and large-diameter studs resulted in a smaller ratio of slippage to *P*/*P_u_*. In this study, the thinner HFRC/OC slabs (150 mm), thinner steel flanges (16 mm), and the presence of bolt hole clearance contributed to a larger ratio of slippage to *P*/*P_u_*. The ratio of *P*/*P_u_* to slippage derived from Equation (9) exhibited lower stiffness, while those from Equations (10)–(12) showed higher stiffness. Figure 13 demonstrates that the ratio of *P*/*P_u_* to slippage from Equation (9) correlated better with the test results. This study suggests that Equation (9) may more accurately predict the relationship between the *P*/*P_u_* ratio and slippage for high-strength, large bolt shear connectors in HFRC/OC. Therefore, Equation (9) could serve as a valuable reference for the design of high-strength, large bolt shear connectors in HFRC/OC.

In this study, only four specimens (NTPT1~2 and HTPT1~2) were prepared for high-strength large through-bolt shear connectors, and the sample size was too small to derive an equation for slip behavior using regression analysis. In a companion paper, the authors [25] conducted tests on ten specimens with through-bolt shear connectors, where the bolt diameter was less than 22 mm. Compared to specimens NTPT1~2 and HTPT1~2, the shear stiffness of these specimens was significantly higher, as no local buckling of the steel flange occurred during the loading process. In the practical design of composite structures, the thickness of the steel flange must be greater than that of the test specimens (16 mm), and the bolt hole clearance is typically grouted. Therefore, data from fourteen specimens were used to determine the slip behavior of high-strength, large through-bolt shear connectors through regression analysis. The authors had previously proposed the slip behavior (Equation (13)) for through-bolt shear connectors (with bolt diameters less than 22 mm) in fabricated HFRC/OC decks. In this paper, the data from fourteen specimens were further analyzed to derive the equation (Equation (14)) of the slip behavior of high-strength large through-bolt shear connectors.

(13) $$P/P_{u}={\left(1-e^{(-0.38+0.206\lambda_{sf}+0.388\lambda_{pf})s}\right)}^{0.88}$$

(14) $$P/P_{u}={\left(1-e^{(-0.39+0.206\lambda_{sf}+0.388\lambda_{pf})s}\right)}^{0.98}$$

The slip behavior of high-strength large through-bolt shear connectors is shown in Figure 13a. He et al. [25], Liu et al. [5], and Zhang et al. [13] overestimated the initial stiffness of high-strength large through-bolt shear connectors with fabricated HFRC/OC decks. This discrepancy might be due to the fact that the load–slip curves from He et al. [25] and Liu et al. [5] did not account for the initial slip caused by the bolt hole clearance after 25 cycles. In the case of Zhang et al. [13], the bolt hole clearance was filled with high-strength grouting material, which likely contributed to the overestimation. Figure 13a demonstrates that the relationship between slippage and the *P*/*P_u_* ratio, as determined using Equation (14) for specimens with fabricated HFRC/OC decks, agreed well with the experimental results. These findings suggest that the equations proposed in this paper can effectively estimate the slippage versus *P*/*P_u_* ratio relationship for through-bolt connectors in fabricated HFRC decks.

(15) $$P/P_{u}=(-0.02+0.206\lambda_{sf}+0.388\lambda_{pf})S\;\;\;\;\;\;\;\;\;\;\;\;\;(P\;\mathrm{less}\;\mathrm{than}\;0.4P_{u})$$

(16) $$P/P_{u}=(1-e^{(-0.379+0.206\lambda_{sf}+0.388\lambda_{pf})S}{)}^{0.98}\;\;\;\;(P\;\mathrm{more}\;\mathrm{than}\;0.4P_{u})$$

The slip behavior of high-strength large bolt shear connectors with grouting pockets is shown in Figure 13b. Wang et al. [35], Liu et al. [5], and Yang et al. [29] also overestimated the initial stiffness of high-strength large bolt shear connectors with grouting pockets. The initial stiffness derived from Equation (9) was higher than that of high-strength large bolt shear connectors with grouting pockets. Therefore, data from six specimens were analyzed to develop an equation for the slip behavior of high-strength large bolt shear connectors with grouting pockets based on the work of Liu et al. [5] and Equation (9). Figure 13b demonstrates that the slip behavior predicted by Equations (15) and (16) agreed well with the experimental results. Thus, the equations (Equations (15) and (16)) proposed in this study may provide valuable references for the design of high-strength large bolt shear connectors with grouting pockets.

## 6. Conclusions

A novel post-installed, high-strength, large bolt shear connector was proposed to transfer shear forces at the interface between the steel girder and fabricated HFRC/OC decks. Eighteen specimens with varying parameters, including different configurations of bolt shear connectors, bolt diameters, single/double nuts, and HFRC/OC materials, were tested to investigate the static performance of post-installed, high-strength, large bolt shear connectors. The following conclusions were drawn:

- Increasing the bolt diameter significantly enhanced the initial stiffness and capacity of post-installed, high-strength, large bolt shear connectors with fabricated HFRC/OC decks. The bolt hole clearance had a notable influence on the initial stiffness, ultimate slip, and capacity of these connectors.
- The incorporation of fibers improved both the capacity and initial stiffness of the connectors. Among the three groups tested, the initial stiffness and capacity of high-strength large through-bolt shear connectors were the lowest, while the other two groups exhibited comparable performance.
- Based on the experimental data, equations for slip behavior and capacity were developed using regression analysis for post-installed, high-strength, large bolt shear connectors with fabricated HFRC/OC decks. These empirical equations were found to provide valuable references for practical applications.
- Future research will focus on investigating the fatigue behavior, creep, and durability of these connectors under environmental exposure, as well as addressing potential limitations related to their implementation.

## Figures and Tables

**Table 1:** Design parameters of specimens.

Specimen	Bolt Diameter (mm)	Configuration of Onnector of Bolt Shear Connector (Figure 1)	Mortar Grade	Concrete Grade	Bolt Grade
NTPT1	27	a	/	C50 (OC)	8.8
NTPT2	30	a	/		
NGPT1	27	b	C80 (80 MPa)		
NPT1	27	d (without stiffness plate)	/		
NGPT2	27	c	C80 (80 MPa)		
NPT2	27	e (without stiffness plate)			
NGPT3	30	c	C60 (60 MPa)		
NGPT4	30	c	C80 (80 MPa)		
NPT3	30	d (without stiffness plate)	/		
NPT4	30	e	/		
HTPT1	27	a	/	C50 (HFRC)	
HTPT2	30	a	/		
HGPT1	30	c	C60 (60 MPa)		
HGPT2	30	c	C80 (80 MPa)		
HPT1	27	d (without stiffness plate)	/		
HPT2	27	e	/		
HPT3	30	d	/		
HPT4	30	e	/		

**Table 2:** Mixture proportion of OC and HFRC (kg).

Concrete	Cement	Water	Gravel	Sand	Fly Ash Particle	Polypropylene	Steel
HFRC (C50)	383	160	1033	533	60	0.15%	1%
OC (C50)	383	160	1033	533	60	0	0

**Table 3:** Physical properties of SF and PF.

Fibers	Density (kg/m^3^)	Average Diameter (mm)	Young’s Modulus (GPa)	Length/Diameter	Tensile Strength (MPa)	Length (mm)
SF	7800	0.7	210	40	600	28
PF	910	0.048	—	167	296	8

**Table 4:** Properties of concrete and mortar.

Specimen	Elastic Modulus/MPa	Compression Strength/MPa	Tensile Strength/MPa
OC (C50)	57.8 (46.2) ^a^	35.9 (30.29) ^b^	4.68
HFRC (C50)	51.8 (41.5) ^a^	34.8 (29.36) ^b^	3.28
Mortar (C60)	38.5	66.2	2.95
Mortar (C100)	40.2	84.1	3.21

^a^ Secant concrete elastic modulus; ^b^ cylinder concrete compressive strength.

**Table 5:** Physical performance of bolts, steel plate, and bar.

Component	Type (mm)	Tensile Strength *f_u_* (MPa)	Yield Strength *f_y_* (MPa)	Elongation Rate (%)
Bolt	*Φ*27	1022	801	16
Bolt	*Φ*30	1061	875	17
Bar	*Φ*12	525	355	29
Steel plate	16	540	435	27

**Table 6:** Initial stiffness, slippage, and shear capacity of specimens NPT1~4 and HPT1~4.

Specimen	NPT				HPT			
	1	2	3	4	1	2	3	4
*P_u_*	221.8	251.3	311.1	327.9	290.8	253.1	331.1	335.6
*S_max_*	7.28	15.19	23.95	11.52	20.55	6.55	13.09	10.16
*K*_0_	174.6	199.1	226.9	298.2	141.3	112.5	275.9	276.3
*K*_1_	398.2	496.1	805.2	1103.7	449.7	565.2	907.8	1192.6

*S_max_* is maximum slippage (mm), *P_u_* is ultimate shear bearing capacity per stud (kN), *K*_0_ is initial stiffness (kN/mm) according to Yang et al. [29], and *K*_1_ is shear stiffness (kN/mm) when the corresponding slippage subtracts the residual slippage after 25 cycles according to Yang et al. [29].

**Table 7:** Shear capacity, stiffness, and ultimate slippage of specimens NTPT1~2, HTPT1~2, HGPT1~2, and NGPT1~4.

Specimen	HGPT		NGPT				HTPT		NTPT	
	1	2	1	2	3	4	1	2	1	2
*P_u_*	318.2	350.1	270	266.1	324.3	345.5	264.3	268.3	219.3	242.1
*S_max_*	24.91	14.39	10.15	30.98	24.05	25.54	12.01	18.6	15.67	16.56
*K_0_*	245.8	229.7	250	222.8	236.4	259.9	91.8	76.1	99.3	59.2
*K_1_*	477	589	326.4	335.6	488.3	511	243.9	215.4	240.6	272.3

**Table 8:** Recommendation capacity formula of through-bolt shear connector.

Reference	Bolt Failure (*P_us_*)	Concrete Failure (*P_uc_*)
He et al. [19]	$P_{us}=0.48(1+0.206\lambda_{sf}+0.388\lambda_{pf})A_{sc}f_{u}$	$P_{U}=0.22{\left(h/d\right)}^{0.4}A_{sc}\sqrt{E_{fc}f_{fc}}$
Zhang et al. [7]	$P_{us}=0.76A_{sc}f_{u}$	$P_{\mathrm{uc}}=0.5A_{sc}\sqrt{f_{c}E_{c}}$
Zhao et al. [25]	$P_{\mathrm{us}}=0.5A_{sc}\sqrt{f_{c}E_{c}}{\left(\frac{d_{b}}{d_{c}}\right)}^{0.6}$	

Note: *h* is effective embedment depth of bolt; *d_b_* is diameter of bolt shank (mm); *d_c_* is diameter of bolt hole (mm); *A_sc_* is cross-sectional area of bolt; *λ_sf_* is SF reinforcement index calculated as *λ_sf_ = V_sf_* (*l_sf_/d_sf_*); *l_sf_*/*d_sf_* is aspect ratio of SF; *V_sf_* is volume fraction of SF; *λ_pf_* is PF reinforcement index, calculated as *λ_pf_* = *V_pf_* (*l_pf_*/*d_pf_*); *l_pf_*/*d_pf_* is aspect ratio of PF; *V_pf_* is volume fraction of PF; *f_c_* is cubic concrete compression strength (Mpa); *E_c_* is concrete elastic modulus (Gpa); *f_u_* is tensile strength of bolt (Mpa); *E_fc_* is elastic modulus of HFRC; *f_c_*, *f_fc_* are compressive strength of NC and HFRC, respectively; *h/d* is ratio of effective embedded depth to bolt diameter—when the ratio is greater than 4, then *h/d* = 1.

**Table 9:** Comparison between test results and calculated result.

Specimen	*P_U_* /(kN)	*P_U_/P_uc_* (Concrete Failure)		*P_U_/P_us_* (Bolt Failure)			
		He et al. [25]	Zhang et al. [13]	He et al. [25]	Zhang et al. [13]	Zhao et al. [31]	*P_U1-MOJ_*
NTPT1	219.3	1.11	0.85	1.53	0.77	0.90	1.03
NTPT2	242.1	1.00	0.76	1.31	0.66	0.80	0.89
HTPT1	274.3	1.42	1.10	1.62	0.97	1.16	1.09
HTPT2	268.3	1.13	0.88	1.28	0.74	0.92	0.87
NC-1	241.8	1.35	0.94	1.51	1.05	0.94	1.02
NC-2	305.5	1.13	0.79	1.26	0.88	0.79	0.85
SFRC-1	248.2	1.32	0.92	1.55	1.07	0.92	1.04
SFRC-2	253.4	1.35	0.94	1.58	1.10	0.94	1.07
SFRC-3	246.7	1.31	0.91	1.54	1.07	0.91	1.04
SFRC-4	242.6	1.29	0.90	1.51	1.05	0.90	1.02
SFRC-5	370.2	1.31	0.91	1.53	1.06	0.91	1.03
SFRC-6	368.1	1.30	0.90	1.52	1.06	0.90	1.03
SFRC-7	376.5	1.33	0.93	1.56	1.08	0.93	1.05
average		1.26	0.90	1.48	0.97	0.92	1.00
Coefficient of variation		0.118	0.078	0.114	0.145	0.084	0.061

**Table 10:** Capacity equations determined for bolt shear connector.

Reference	Concrete Failure (*P_uc_*)	Steel Failure (*P_us_*)
Zhang et al. [33]	$0.7A_{sc}\sqrt{f_{c}E_{c}}$	$0.62A_{sc}f_{u}$
Pathirana et al. [1]	$0.31d_{b}^{2}\sqrt{f_{c}E_{c}}$	$0.63d_{sc}^{2}f_{u}$
Yang et al. [29]	/	$0.8A_{sc}f_{u}$

**Table 11:** Comparison of calculated values and test results of bolt connector.

Specimen	*P_U_* /(kN)		*P_U_/P_uc_* (Concrete Failure)			*P_U_/P_us_* (Shear Connector Failure)			
		Pathirana et al. [1]	Zhang et al. [33]	*P_U_/P_u3-MOJ_*	*P_U_/P_u4-MOJ_*	Pathirana et al. [1]	Yang et al. [29]	Zhang et al. [33]	*P_U_/P_u2-MOJ_*
NPT1	211.8	1.10	0.75	0.75	1.10	0.71	0.71	0.91	0.89
NPT2	251.4	1.31	0.89	0.89	1.31	0.84	0.84	1.08	1.03
NPT3	311.1	1.31	0.90	0.90	1.31	0.81	0.81	1.05	1.04
NPT4	328	1.38	0.94	0.94	1.38	0.85	0.85	1.10	1.08
HPT1	290.8	1.50	1.02	1.03	1.52	0.97	0.97	1.25	1.07
HPT2	253	1.30	0.89	0.90	1.32	0.84	0.84	1.09	0.95
HPT3	331.1	1.38	0.94	0.95	1.40	0.86	0.86	1.11	1.00
HPT4	335.6	1.40	0.96	0.97	1.42	0.87	0.87	1.13	1.01
B7	308.7	1.03	0.70	0.70	1.03	0.81	0.81	1.05	1.01
B8	286.6	0.96	0.65	0.65	0.96	0.76	0.76	0.98	0.94
B9	256.8	1.10	0.88	0.88	1.10	0.85	0.85	1.11	1.16
Average		1.25	0.87	0.87	1.26	0.83	0.83	1.08	1.00
CV		0.167	0.11	0.113	0.173	0.063	0.063	0.082	0.061

**Table 12:** Comparison of test results and calculated values of bolt shear connector with grouting pocket.

Specimen	*P_U_* /(kN)	*P_U_/P_uc_* (Concrete Failure)				*P_U_/P_us_* (Shear Connector Failure)				
		Pathirana et al. [1]	Zhang et al. [33]	*P_U_/P_u3-MOJ_*	*P_U_/P_u4-MOJ_*	Pathirana et al. [1]	Yang et al. [29]	Zhang et al. [33]	*P_U_/P_u1-MOJ_*	*P_U_/P_u2-MOJ_*
NGPT1	270	1.37	0.94	0.94	1.37	1.16	0.90	0.90	1.02	1.10
NGPT2	266.1	1.35	0.92	0.92	1.35	1.15	0.89	0.89	1.00	1.10
NGPT3	324.3	1.33	0.91	0.91	1.33	1.09	0.84	0.84	0.95	1.08
NGPT4	345.5	1.42	0.97	0.97	1.42	1.16	0.90	0.90	1.01	1.09
HGPT1	318.2	1.36	0.93	0.91	1.34	1.07	0.83	0.83	0.79	0.94
HGPT2	350.9	1.47	1.01	1.00	1.47	1.18	0.91	0.91	0.87	1.00
Average		1.38	0.94	0.94	1.38	1.13	0.88	0.88	0.94	1.05
CV		0.048	0.035	0.033	0.05	0.041	0.031	0.031	0.084	0.051

## Data Availability

The original contributions presented in this study are included in the article. Further inquiries can be directed to the corresponding author.
